# THE EPIDEMIOLOGY OF SOCIAL ISOLATION AND LONELINESS OF FAMILY AND UNPAID CAREGIVERS OF OLDER ADULTS

**DOI:** 10.1093/geroni/igae098.0804

**Published:** 2024-12-31

**Authors:** Yiqing Qian, Mary Louise Pomeroy, Martha Saylor, Katherine Ornstein

**Affiliations:** Johns Hopkins University, Baltimore, Maryland, United States; Johns Hopkins University, Baltimore, Maryland, United States; Johns Hopkins University, Baltimore, Maryland, United States; Johns Hopkins University, Baltimore, Maryland, United States

## Abstract

The experience of caring for older relatives and friends can be socially isolating. Family and unpaid caregivers (“caregivers”) may encounter barriers to social participation (e.g., caregiving duties) and unmet needs in support. Given limited epidemiological evidence of social isolation (SI) and loneliness in this population in the U.S., we reported the prevalence and correlates of SI and loneliness in the National Study of Caregiving Round 12 (n=2,119). Most were females (62%), non-Hispanic white (69%), and had a high school education (91%). Most cared for a parent/parent-in-law (46%). Caregivers provided an average of 70 hours of care/month. On a 4-item SI scale ranging from 0 (high SI) to 4 (low SI), the mean score was 2.6. About 13% of caregivers were categorized as socially isolated. About 33% reported feeling lonely “some days,” “most days,” or “every day.” Logistic regression showed female caregivers (OR: 0.56[0.37,0.86]) and those with lower education levels (OR[college vs below high school]: 0.14[0.06,0.33]) were less likely to be socially isolated vs not isolated, controlling for other socio-demographic (e.g., age) and caregiving characteristics (e.g., hours). Adjusted ordinal logistic regression further showed SI levels among care recipients were associated with SI levels among caregivers (OR: 1.66[1.38,2.00]). Null associations were found in parallel analyses on loneliness. This study highlights the heterogeneity of SI among caregivers of older adults and the necessity of examining the shared experiences with care recipients. More comprehensive loneliness instruments in public health surveillance may advance the understanding of experiences and impacts of SI and loneliness in caregiving.

